# Thoratec Centrimag VAD for cardiogenic shock – a developing country experience

**DOI:** 10.1186/1749-8090-8-S1-O156

**Published:** 2013-09-11

**Authors:** M Villavicencio, V Rossel, R Larrea, J Peralta, E Larrain, J Lim, P Rojo, E Donoso, F Gajardo, M Hurtado

**Affiliations:** 1 Cardiopulmonary Transplant Service, Instituto Nacional del Tórax, Santiago, Chile

## Background

Circulatory support is usually not available in developing countries due to cost restrictions. Long-term devices are almost always not affordable for our health system and Thoratec Centrimag® may be an alternative. We report our experience in the treatment of cardiogenic shock with this intermediate-term device.

## Methods

Thoratec Centrimag® was used in 22 cardiogenic shock patients. All were INTERMACS class I and all but one had multiorgan failure. Seventeen (77%) were male, mean age 41+13 years. Etiology was ischemic in 11(50%), dilated in 4(22%), and other etiology in 7(32%). Nine (41%) had previous cardiac arrest with a mean arrest time of 18+16 min. Circulatory support was biventricular in 18(82%), univentricular 4(18%), and in 6(27%) an oxygenator was interposed within the outflow line until respiratory recovery. Patients with veno-arterial ECMO configurations were excluded.

## Results

Bridge-to-transplant or recovery was obtained in 14 out of 22 (64%). Mean support time 44 days, range 1-292 days. Eight patients (36%) were supported for more than 4 weeks. Thirty-day post-implant survival was 73% (16 patients). Post-implant complications were re-exploration for bleeding 7(32%), neurologic dysfunction 3(14%), pneumonia 8(36%), and renal failure with dialysis in (32%). Eleven (50%) patients are in NYHA class I after a mean follow-up time of 32+6 months. Kaplan Meier one-year survival was 56+11%. Eleven out of 12 (92%) bridged-to-transplant are in NYHA functional class I with normal biventricular function. One patient died 3 days post-transplant due to inflammatory response. Two patients were bridged-to-recovery. One is in NYHA class I and the other died due to non-compliance.

## Conclusion

Thoratec Centrimag® is useful to provide intermediate-term circulatory support for cardiogenic shock and multiorgan failure in a developing country. Support time longer than 4 weeks is feasible. A multidisciplinary approach is needed since morbidity is common.

